# Propagation of intense laser pulses in plasma with a prepared phase-space distribution

**DOI:** 10.1038/s41598-022-24664-x

**Published:** 2022-11-27

**Authors:** Devki N. Gupta, Samuel R. Yoffe, Arohi Jain, Bernhard Ersfeld, Dino A. Jaroszynski

**Affiliations:** 1 Department of Physics and Astrophysics, University of Delhi, Delhi, 110 007 India; 2 Department of Physics, SUPA and University of Strathclyde, Glasgow, G4 0NG UK

**Keywords:** Plasma-based accelerators, Free-electron lasers

## Abstract

Optimizing the laser wakefield accelerator (LWFA) requires control of the intense driving laser pulse and its stable propagation. This is usually challenging because of mode mismatching arising from relativistic self-focusing, which invariably alters the velocity and shape of the laser pulse. Here we show how an intense pre-pulse can prepare the momentum/density phase-space distribution of plasma electrons encountered by a trailing laser pulse to control its propagation. This can also be used to minimize the evolution of the wakefield thus enhancing the stability of the LWFA, which is important for applications.

## Introduction

The laser-plasma accelerator^1–4^ has advanced to the stage where control of its beam parameters^5–11^ and stability are the main development challenges. Achieving high quality electron beams in laser wakefield accelerators requires stable propagation of the driving laser pulse^12–18^. This is usually challenging because of mode mismatching arising from relativistic self-focusing. Here we present a numerical study to show the wakefield properties can be altered by the presence of an intense pre-pulse. The role of the pre-pulse is to prepare the phase-space distribution of plasma electrons prior to the arrival of an intense trailing driver pulse. This enables the driver pulse to produce a well-matched plasma channel that can control or minimise wakefield evolution. This type of channelling differs from that of preformed plasma channels, which require excellent alignment between channel and laser (driver) pulse to be guided^19,20^. Controlling the intensity of laser pulses is an essential step in developing useful wakefield accelerators and compact radiation sources^21–23^. Stable channels are necessary for developing next generation compact plasma undulators for compact synchrotron sources^24,25^ and free-electron lasers^26,27^. Wide availability of ultra-compact accelerators and radiation sources could transform the way science is done^21,28^.

Particle acceleration in the laser wakefield accelerator (LWFA)^1^ is due to the ponderomotive force of an intense, ultra-short duration driver pulse, which separates electrons from ions. The resulting ultra-high electric field, which can exceed 100 GV/m, is several orders of magnitude larger than possible in conventional accelerators. Displaced electrons follow trajectories that define a thin sheath surrounding the ion-filled plasma bubble and converge at the rear of the bubble. When their velocities exceed the velocity of the wake they are injected into the bubble and rapidly accelerated to high energies^5,6,11^ with excellent properties^7–10^. They can also be used as compact all-optical sources of brilliant X-ray^24^ and gamma-ray^25^ photons that have potential applications including spectroscopy and imaging for material sciences, warm dense matter studies for laboratory astrophysics and fusion, and for the biology and health sciences^29–31^. Ultra-short pulse LWFAs are being investigated as drivers of synchrotron sources^22^, free-electron lasers^21,23,32^ and ion channel lasers (ICLs)^26,27^, and for very high energy electron (VHEE) radiotherapy^33–35^, pulsed radiolysis^36^ and medical imaging^30,31^.

Many applications require high-quality electron bunches with low emittance, ultra-short duration and high peak current. Electron bunch properties depend on maintaining field uniformity^21,28^. The bubble evolution directly influences self-injection^10,37,38^. Laser^39^ and plasma^37^ variations which cause bubble evolution, result in time-varying electrostatic fields that have a direct impact on electron bunch quality^40^. Stable propagation of the driver pulse is required to minimise bubble variations to ensure persistent acceleration and high quality beams.

The propagation of intense laser pulses is a highly nonlinear process; the laser pulse and the plasma medium are mutually modified during propagation. Stable propagation of the intense driving laser pulse ensures stable wakefield evolution. Variations of the wakefield and evolution of bubble shape and velocity strongly affect electron bunch parameters, such as charge, emittance and energy spread^41^. It can also cause transverse oscillations of the bubble and collective transverse oscillations of the electron beam^42,43^ that increases the beam divergence and transverse emittance, and can reduce the dephasing length, which ultimately reduces the maximum energy gain.

In this article, we present a double-pulse method of preparing the electron momentum distribution of plasma in advance of the arrival of an intense trailing driver pulse. The short-duration pre-pulse enables the trailing driver pulse to form its own self-aligned channel. Optimum formation is achieved when the time delay between the two pulses is slightly less than the plasma period, $t_d \lessapprox \lambda _p/c$. The moderately intense, leading, or chaperone, pre-pulse excites a weakly-nonlinear plasma wave, while remaining below the threshold for wavebreaking. It does not directly produce a guiding structure for the trailing driver pulse, but prepares the plasma by producing converging streams of electrons that are subsequently deflected by the ponderomotive force of the (trailing) driver pulse to produce a narrow, well-defined parabolic plasma channel. This self-aligns the waveguide over extended propagation lengths. The driver pulse initially undergoes relativistic self-focusing until it matches the narrow density channel; negative feedback ensures stable matching and high coupling efficiency. The chaperone pre-pulse stimulates the driver pulse to form a channel, which ensures control over its evolution as well as the stability of the wakefield. It can be used for a variety of purposes, for example to suppress spontaneous self-injection and to control the electron bunch properties. As the wake is not resonantly excited by the driver pulse, electrons do not initially have the necessary phase to be self-injected. However, the amplitude of the driver laser pulse is maintained by the density channel, which results in stable acceleration.

## Results

**Figure 1:**
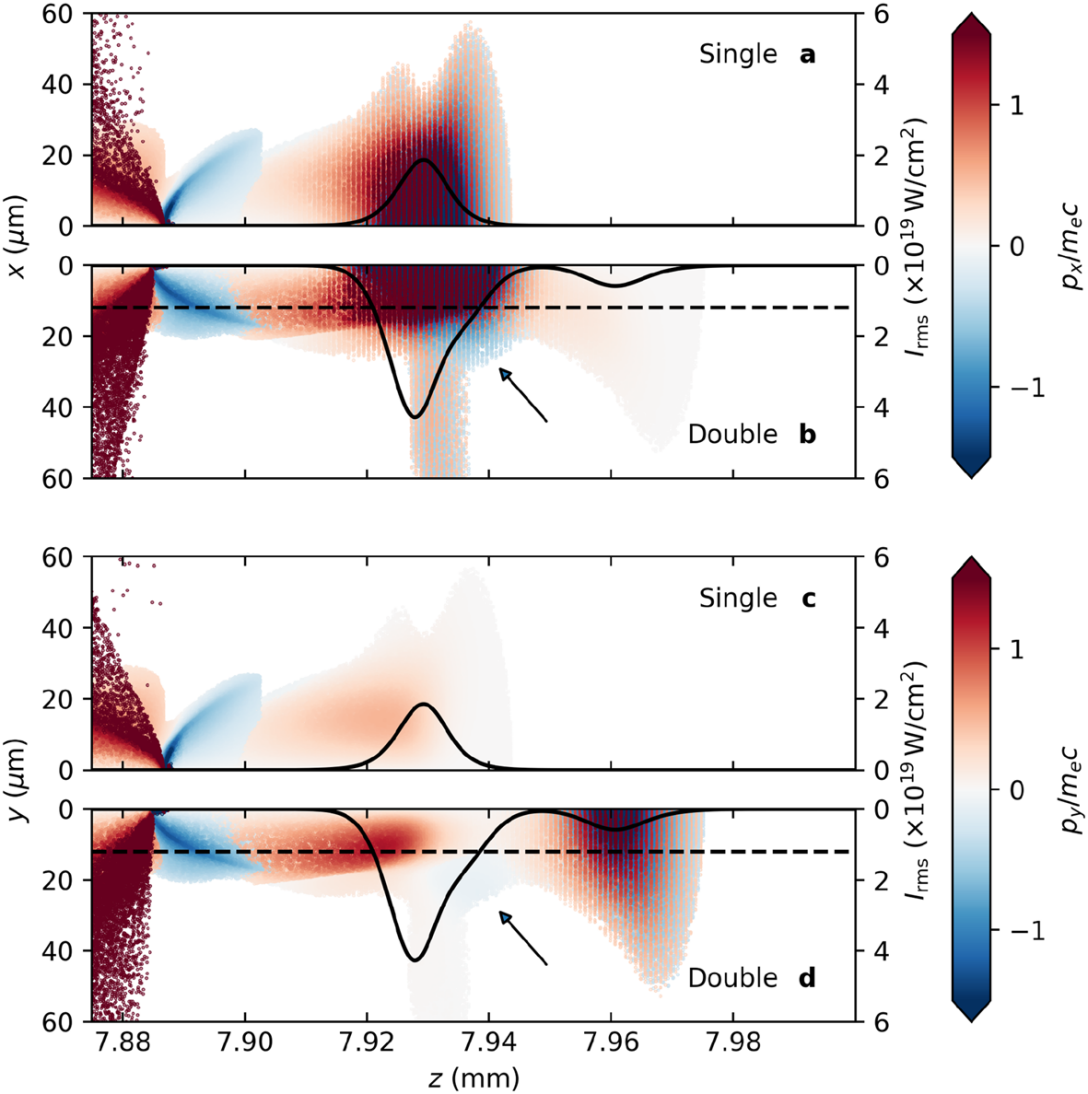
Comparison of electron distribution with and without the pre-pulse. Distribution of normalized transverse momentum, $p_x/m_ec$, for the plasma electrons (**a**) without and (**b**) with the pre-pulse. The on-axis intensity of the driver pulse and pre-pulse are shown by the black bold line. Similar figures for $p_y/m_ec$ are shown in part (**c**) for the single pulse and (**d**) for the double pulse configurations. The converging electrons indicated by the arrows lead to the production of a co-moving parabolic density channel. The dashed lines in parts (**b**) and (**d**) indicate the fitted parabolic channel radius, as shown in Fig. 3. (Note that the values of the color bars have been truncated for clarity).

**Figure 2:**
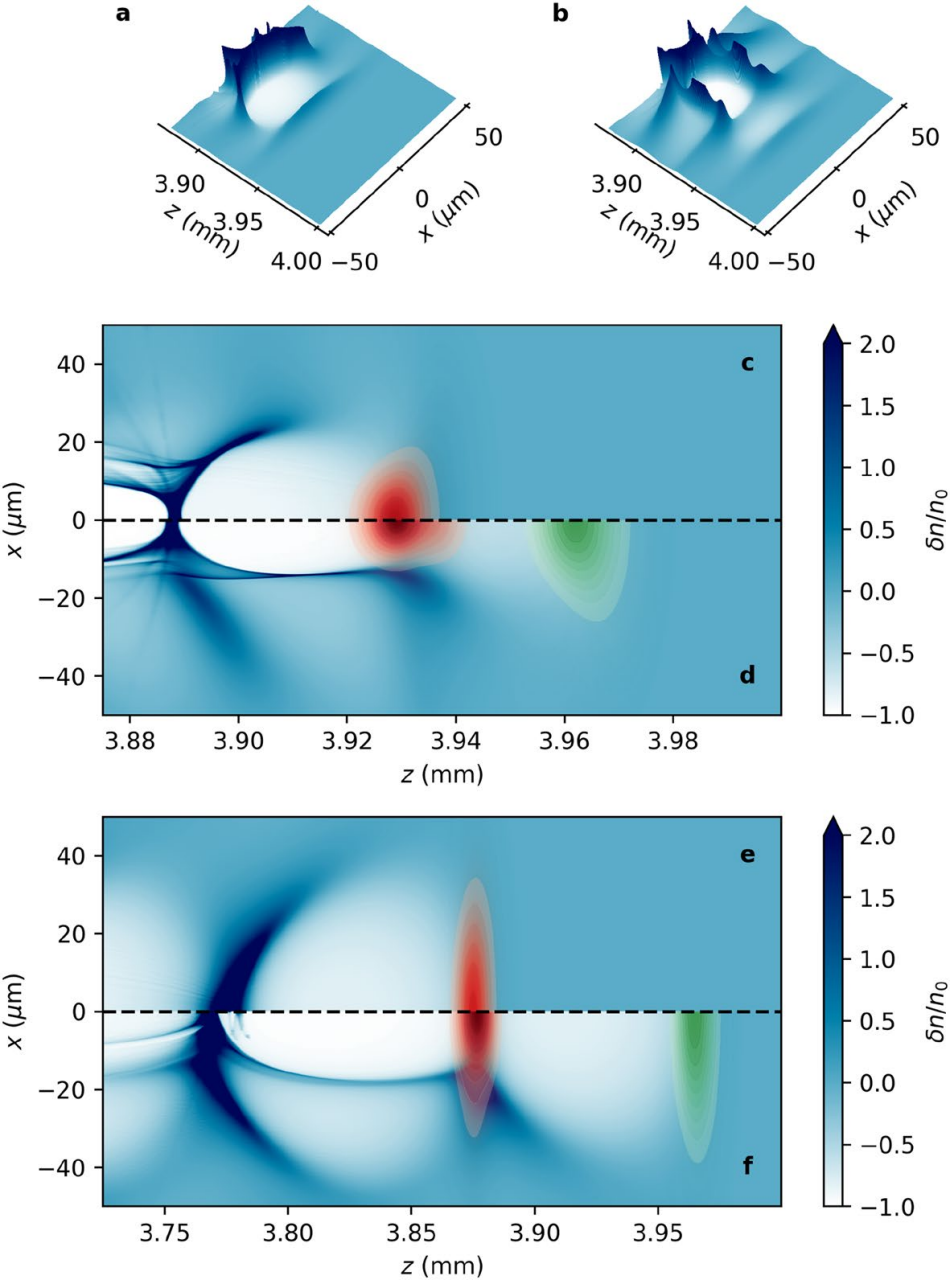
Simulation results demonstrate the formation of the channel stimulated by the pre-pulse. The electron density ($n_e$) modulation $\delta n/n_0 = (n_e - n_0)/n_0$ (with ${n_0 = 7.17 \times 10^{17}}$ cm$^{-3}$) visualized (**a**) without and (**b**) with a pre-pulse. (Note that the vertical axis has been truncated at $\delta n/n_0 = 2$ for clarity.) The electron density and the driver pulse intensity (red) are shown in part (**c**) without, and in part (**d**) with, the pre-pulse laser (green). Similar production of a density channel in lower density plasma ($n_0 = 1\times 10^{17}$ cm$^{-3}$) is illustrated by parts (**e**) and (**f**), without and with the pre-pulse, respectively. Parts (**a**)–(**f**) use the same colour scale.

Particle-in-cell (PIC) simulations have been performed (see "Methods' section) to evaluate the influence of the leading pre-pulse. We compare the double pulse configuration with the case without the pre-pulse. We also consider the case for a single pulse that has the combined energy of the driver pulse and pre-pulse.

As described above, the purpose of the pre-pulse is not to directly produce a plasma channel to guide the driver pulse, but to prepare the plasma to facilitate the production of a channel by the driver pulse (see Supplementary Fig. S1). Figure 1 compares the phase-space distribution of plasma electrons with and without the pre-pulse. In the double pulse case, the main driving laser pulse interacts with electrons converging with inward momentum at the rear of the pre-pulse wake to produce a well-defined parabolic plasma channel. The parabolic channel radius, $r_c$, is indicated by the dashed line, and clearly separates the region of converging electrons (indicated by the arrow) from those oscillating in the intense laser field. The phase-space distribution is different in *x*- and *y*-direction due to the laser polarization of pre-pulse and main pulse in the *y*-direction and *x*-direction, respectively. The delay is fine-tuned to ensure that electrons displaced by the pre-pulse interact with the driver pulse as they converge at the back of the pre-pulse wake, where they form a co-moving parabolic density channel (Fig. 2b) in which the driver pulse propagates. In contrast, the density modulation produced by a single driver pulse, as illustrated in Fig. 2a, shows the familiar wake without evidence of a channel structure. In Fig. 2c, the electron density distribution does not create structure capable of ensuring stable propagation of the single driving laser (red), whereas in Fig. 2d a waveguide stimulated by the pre-pulse (green) is evident in the region of the driver pulse (red). In the latter case, the driver pulse maintains a high peak intensity and a narrower beam waist over a long distance. This demonstrates the formation of a channel that enables stable propagation of an intense laser pulse in plasma with density $7.17\times 10^{17}$ cm$^{-3}$. A similar plasma channel can be created in $1\times 10^{17}$ cm$^{-3}$ plasma, as shown in Fig. 2e and f with initial $a_0 = a_1 = 2.5$, where $a_0$ and $a_1$ are the initial normalized amplitudes of the pre-pulse and driver pulse, respectively. Due to the increased resources required to perform these simulations at lower density, the latter has not been fully optimized. However, channel formation is clearly demonstrated at lower densities.

**Figure 3:**
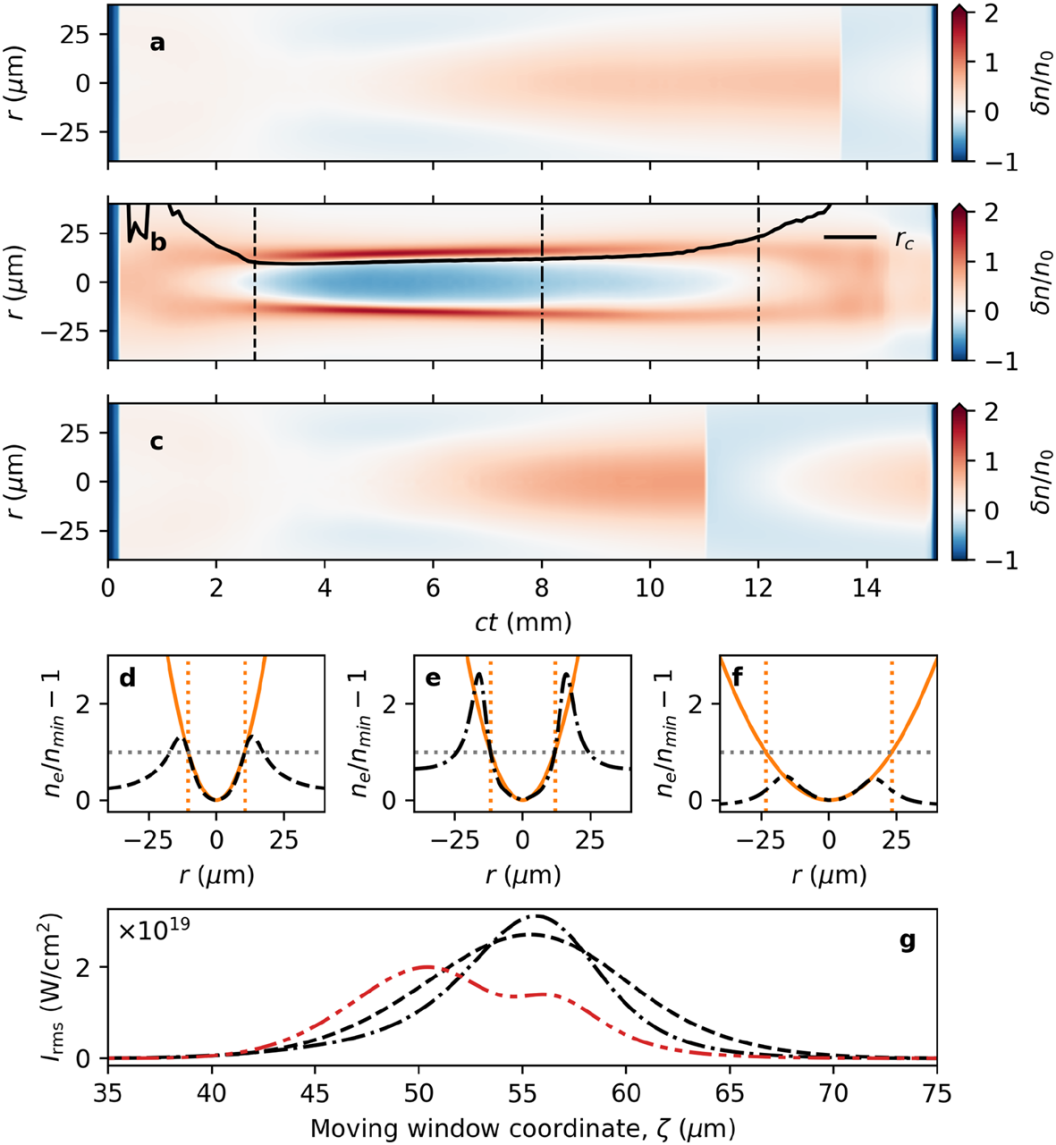
Evolution of the transverse electron density profile at the laser peak. Evolution of the transverse electron density profile, determined at the position of peak intensity of the driver pulse, for the (**a**) single driver pulse; (**b**) pre-pulse and driver pulse; and (**c**) higher-intensity single driver pulse configurations. Panel (**b**) demonstrates the production of a persistent plasma channel, with steep walls and a low density core. The maximum channel height at the position of peak laser intensity is $\delta n/n_0 = 2$. Lineouts of the density profile at $ct = 2.7$ mm (- - -), $ct = 8$ mm ($\cdot$ −), and $ct = 12$ mm ($\cdot$ $\cdot$ −) from part (**b**) are shown in panels (**d**)–(**f**). For the double pulse case, the corresponding parabolic channel profile is plotted in orange, indicating that a parabolic density channel is produced and maintained over a significant distance. The measured channel radius is (**d**) $r_c = 10.4\,\upmu$m, (**e**) $r_c = 11.8\,\upmu$m, and (**f**) $r_c = 23.3\,\mu$m. Part (**g**) shows the on-axis intensity (as a function of the moving window coordinate, $\zeta = z - ct$) for the higher-intensity single pulse at the three positions, as above, illustrating the development of a second peak that becomes the maximum, causing the discontinuity observed in parts (**a**) and (**c**).

Figure 3 presents the transverse density profile (averaged over half a laser wavelength) at the position of maximum intensity of the driver pulse, for cases (a) without the pre-pulse; (b) with the pre-pulse; and (c) a single higher-intensity driver pulse. In the single pulse cases, Figs. 3a and c, the density peaks on the laser axis, and no channel structure is observed. In contrast, Fig. 3b demonstrates the production of a density channel with steep walls and reduced density at the centre, which persists while the laser pulse propagates from 2 mm to 12 mm. The solid curve shows the channel radius, $r_c$, for a parabolic channel of the form $n_e = n_\text {min} ( 1 + r^2/r_c^2 )$, where $n_\text {min}$ is the density at the centre of the channel. The lineouts (cross-section) of the transverse density profile at 2.7 mm (dashed), 8 mm (dot-dashed), and 12 mm (dot-dot-dashed) are shown in panels Fig. 3d–f, along with the corresponding parabolic profile in orange. We observe that the channel density profile is well described by a parabola and that the structure accompanies the driver pulse over an extended length.

**Figure 4:**
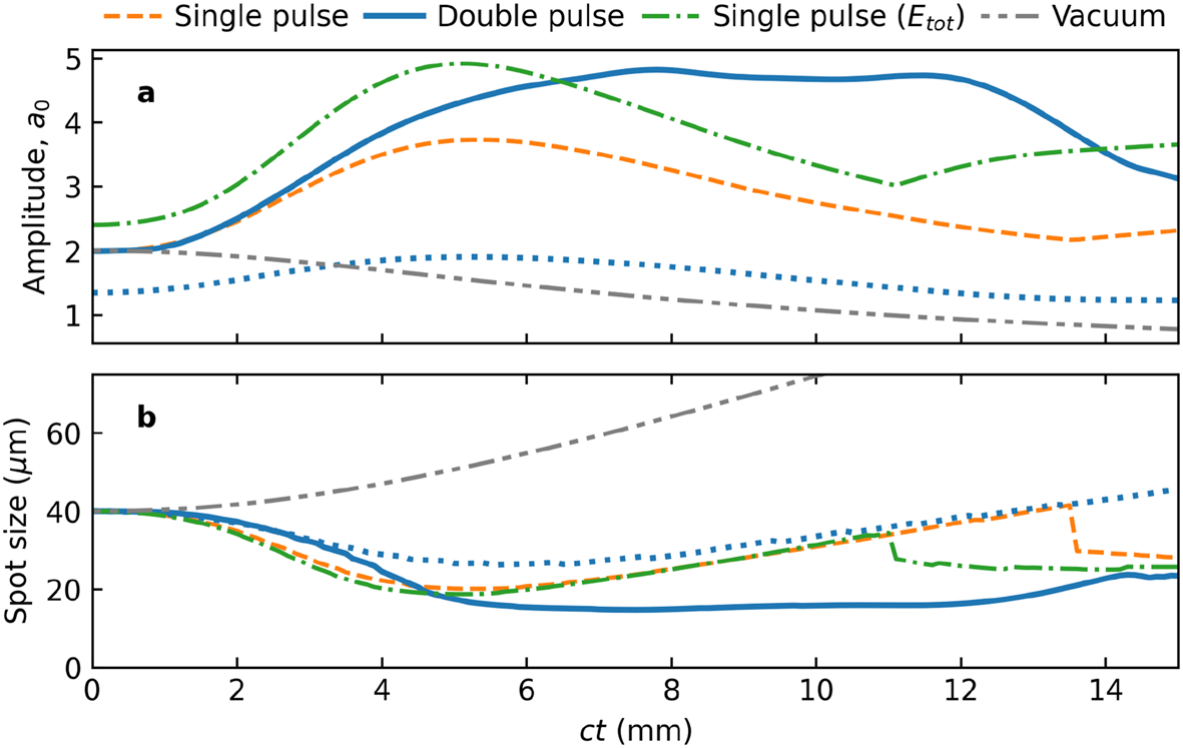
Comparison of driver pulse evolution for the single and double pulse cases. Evolution of the driver pulse (**a**) amplitude and (**b**) waist, for the single driver pulse (- - -), double pulse (**—**), and higher-intensity single driver pulse ($\cdot$ −) cases. A region of stable driver pulse guiding is identified for the double pulse case between 5 mm and 12 mm. Vacuum propagation for the (initial) driver pulse ($\cdot$ $\cdot$ −) is shown for comparison, along with the evolution of the pre-pulse ($\cdots$). Data for the single pulse LWFA (- - -) originally appeared in Yoffe et al.^44^.

The production of a co-moving parabolic density channel suggests that it acts as a waveguide for the driver pulse. Figure 4a shows the temporal evolution of the amplitude with and without the pre-pulse, and for vacuum propagation. The driver pulse alone is observed to self-focus in the plasma before diffracting. However, it has a stable amplitude and pulse waist when the pre-pulse is present (see Fig. 4b). This indicates a matched pulse, confirming that the parabolic density channel acts as a well-matched waveguide. The matched laser spot size^13^ for the instantaneous plasma channel density and normalized laser amplitude at 7 mm is $W_M = 13.9$ $\mu$m, while the value of the laser spot is $r_l = 14.9$ $\mu$m. This is in good agreement, considering that it does not account for relativistic effects or the electron momentum distribution generated by the pre-pulse. Data presented in Fig. 4 for the single pulse LWFA (- - -) originally appeared in Yoffe et al.^44^.

The discontinuities observed for the single pulse cases (Figs. 3a,c and 4a,b) are artefacts due to sudden jumps in the position of the intensity maximum. As the rear of the driver pulse is focused and a second peak grows to a larger intensity. This is illustrated in Fig. 3g for the high-intensity single driver pulse.

**Figure 5:**
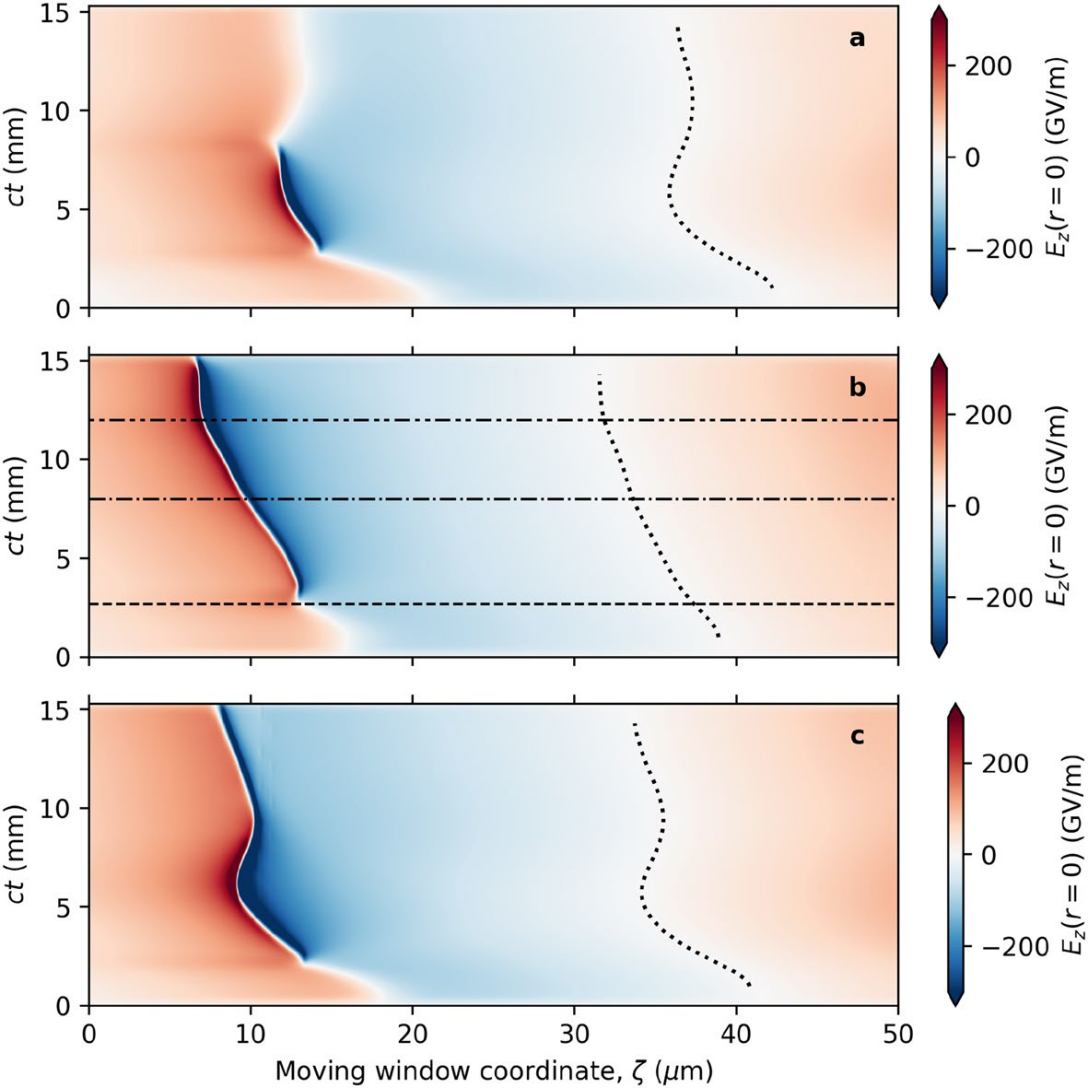
Evolution of the longitudinal electric field. Temporal evolution of the on-axis longitudinal electric field for the (**a**) single pulse; (**b**) double pulse; and (**c**) higher-intensity single pulse configurations, shown in the moving window, $\zeta = z - ct$ (a vertical line would represent motion at *c*). Horizontal lines indicate the propagation distances 2.7 mm, 8 mm and 12 mm highlighted and discussed in Fig. 3. Dotted lines indicate the position of the dephasing point.

One consequence of stable propagation of the intense driver pulse is a significant improvement in the stability of the plasma wave: the bubble maintains a consistent shape and velocity, minimizing time-dependent fields that would otherwise lead to increases in energy spread and emittance^40^. Figure 5 shows the time evolution of the on-axis longitudinal electric field, $E_z$. Figures 5a and c show the field for the single driver pulse cases, where the centre (dotted line) and back of the bubble are observed to fluctuate significantly. In Fig. 5a we also observe that the longitudinal field is only strong over a short propagation distance. However, it is observed in Fig. 5b that with the chaperone pre-pulse, the strong field segment and the bubble velocity are much more stable.

**Figure 6:**
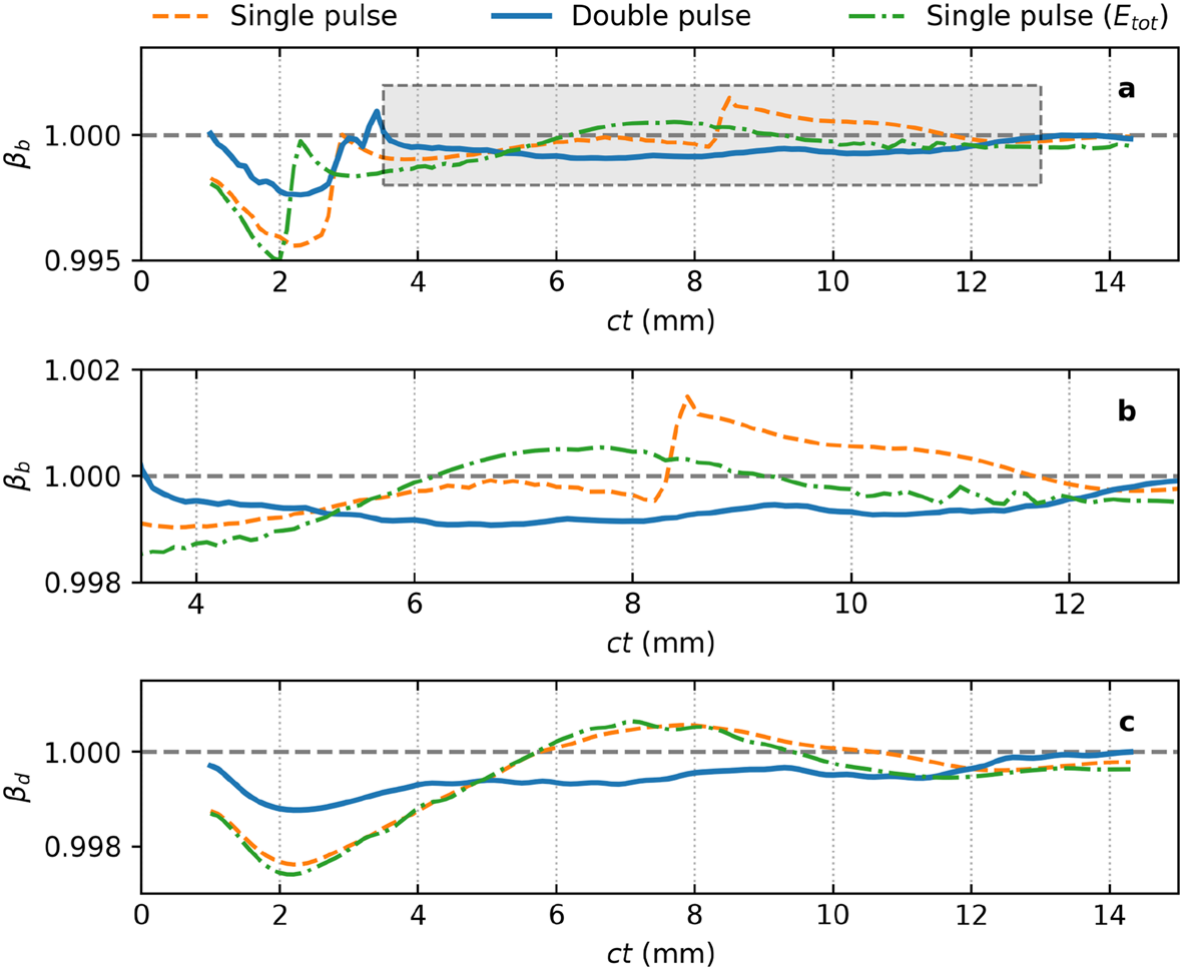
Evolution of the bubble velocity. Part (**a**) shows the variation of the (normalized) velocity of the back of the bubble, $\beta _b$ for the single pulse (- - -), double pulse (**—**), and higher-intensity single pulse ($\cdot$ −) configurations, with the highlighted region expanded in part (**b**). Part (**c**) shows the variation of the (normalized) velocity of the dephasing point at the centre of the bubble, $\beta _d$. Data presented in Fig. 6c for the single pulse LWFA (- - -) originally appeared in Yoffe et al.^44^.

Figure 6a shows the evolution of the velocity of the back of the bubble (defined as the zero-crossing of the longitudinal electric field) with the shaded region enlarged in Fig. 6b. The velocity of the dephasing point is presented in Fig. 6c. Data presented in Fig. 6c for the single pulse LWFA (- - -) originally appeared in Yoffe et al.^44^. The (dimensionless) bubble velocity, $\beta _b$, and dephasing point velocity, $\beta _d$, are observed to vary significantly for the two cases without the pre-pulse, becoming superluminal. The presence of the pre-pulse and stimulating the channel formation for the driver pulse stabilizes both the laser evolution (such as its group velocity^44^ and therefore dephasing point) and the wakefield it generates. The increased stability of the wake using a double pulse will help with developing high-quality laser wakefield accelerators (LWFAs), and also for new applications of ion channels such as for use in the ion channel lasers (ICLs)^27^, which require the electron bunch to remain at the dephasing point to minimise energy variations. Matching the electron bunch velocity to that of the dephasing point is only feasible if the latter varies slowly, which can be achieved using the pre-pulse to ensure that electron bunches injected with the correct velocity and phase will remain near the dephasing point for an extended period.

## Conclusions

We have demonstrated how the introduction of a lower intensity chaperone pre-pulse in the LWFA can prepare the electron phase-space so that a trailing driver pulse produce its own co-moving plasma channel. For the parameters investigated here, we find that a pre-pulse with a delay of 0.85$\lambda _p/c$ (informed by nonlinear 1D theory for, and simulation of, its wake) prepares the plasma electron phase-space for a focused high-intensity ($I_\text {rms} > 4.5\times 10^{19}$ W/cm$^2$) 430 TW pulse to form a parabolic density channel that ensures stable propagation over nearly a centimetre in low density plasma, where it is usually difficult to produce suitable plasma waveguides^11^. The scheme presented here does not require alignment or synchronization, which are usually challenging when using preformed plasma waveguides. The driver pulse energy coupling efficiency into the channel at 2.7 mm is 98.7%, with an overall transmission efficiency of 89.3% observed when the laser propagates from 2.7 mm to 12 mm (including the loss of energy to create the channel and wake). The scheme could be optimised by varying the laser pulse parameters, tuning the inter-pulse delay, or guiding the pre-pulse.

It is important that the pre-pulse is well matched to the plasma or guided so that it persists over the required interaction length. Guiding the pre-pulse using a preformed channel does not negate the stability benefits offered by the double pulse configuration presented here. For the results obtained here, we found that the best channel and most stable propagation are observed when the pre-pulse amplitude $a_0 > 1.7$. To extend the scheme to lower densities, while matching the pre-pulse parameters for stable propagation, it will be necessary to increase its width and power to remain above the critical power to sufficiently alter the phase-space distribution for the driver pulse. The channel must also be wider to ensure that the group velocity of the driver pulse is matched to that of the pre-pulse.

The stabilizing effect of the chaperone pre-pulse significantly improves the overall stability of the plasma wake. The double-pulse configuration should contribute to the development of controllable LWFAs for high-energy, high-quality electron bunches for a wide range of applications, such as drivers of secondary radiation sources. We also note that the stability of the ion cavity, driver pulse amplitude and plasma density can be used to control the velocity of the dephasing point, making this an attractive ion channel for an ICL or plasma wiggler. To aid the design of experiments to verify the mechanism described here, we note that similar results are obtained using parallel polarizations for the pre-pulse and driver pulse.

## Methods

Computer simulations are performed using the open source code FBPIC^45^, which is a quasi-3D PIC code that uses cylindrical geometry and azimuthal modal decomposition to represent the 3D domain. This significantly reduces the computational resources required, especially for large domains, while retaining important 3D effects. The spectral solver used by the code reduces numerical artifacts, such as noise, dispersion and numerical Cherenkov radiation. Simulations have been performed for various laser intensities for single and double laser pulses, using a moving window of size $z\times r = 156.25\lambda _0 \times 125\lambda _0$ with $3126 \times 1000$ grid points propagating at the speed of light. From the geometry of the laser and plasma, it is expected that only azimuthal modes $m = 0,1$ will be important. The simulations presented here use azimuthal modes $m = 0,1,2$ with (3,2,12) particles per cell in ($z,r,\theta$), but results are almost identical to the case using two modes and fewer particles per cell.

We first consider the wakefield generated by a single Gaussian-profile driver pulse with an initial normalized amplitude $a_1 = 2$, which is defined from the electric field, $\textbf{E}$, as $a = e\lambda |\textbf{E}|/(2\pi m_e c^2)$, where $\lambda _1 = 800$ nm is the central laser wavelength (pulse intensity $I_\text {rms} = 8.55\times 10^{18}$ W/cm$^2$). The driver pulse has full-width at half-maximum (FWHM) intensity duration $\tau _1 = 45$ fs and focal spot size (at $1/e^2$ intensity) $r_1 = 40\,\mu$m (energy 10.29 J). Simulations are then repeated adding a pre-pulse (with the same wavelength, duration and spot size as above) but with initial normalized amplitude $a_0 = 1.35$ (intensity $I_\text {rms} = 3.90\times 10^{18}$ W/cm$^2$, energy 4.69 J). Plasma waves are resonantly excited^46^ when the delay length is equal to the plasma wavelength, $\lambda _p$. However, here we choose a delay $ct_d = 0.85\lambda _p$ so that the position of the driver pulse is in front of the density maximum of the (unperturbed) pre-pulse wake. To clearly distinguish the pre-pulse from the driver pulse, the polarizations are chosen to be orthogonal. However, similar results are obtained for pulses with the same polarization. Finally, a third case is considered using a single Gaussian-profile driver pulse containing the total energy of the driver and pre-pulse from the double pulse case above, $E_\text {tot} = 14.98$ J with an initial normalized amplitude $a_1 = 2.413$ (intensity $I_\text {rms} = 1.24\times 10^{19}$ W/cm$^2$). The plasma is taken to be a 15 mm flat-top plateau with electron number density $n_0=7.17\times 10^{17}$ cm$^{-3}$ and 100 μm sine-profile ramps. Laser pulses are focused at the top of the plasma density upramp. These parameters are chosen for a proof-of-principle demonstration, which could be further optimized.

As a guide to finding a starting point for the delay optimization, we use a nonlinear 1D theory^47,48^ with the plasma and pre-pulse parameters. We consider a laser pulse propagating along the *z*-axis with Gaussian profile defined by $a^2(\zeta ) = a_0^2\ \exp (-\zeta ^2/(c\tau _0)^2)$, where ${\zeta =z-v_g}t$ with $v_g$ the laser group velocity and $\tau _0$ is the laser pulse duration. The normalized wake potential $\phi = e\Phi /(m_e c^2)$ is obtained by numerically solving the second-order nonlinear differential equation for the nonlinear regime:

1 $$\begin{aligned} \frac{\partial ^{2} \phi }{\partial \zeta ^{2}}=k_{p}^{2} \gamma _{p}^{2}\left[ \beta _{p}\left( 1-\frac{1+a^{2}}{\gamma _{p}^{2}(1+\phi )^{2}}\right) ^{-1 / 2}-1\right] , \end{aligned}$$

where $\gamma _p=(1-\beta _p^2)^{-1/2}$ is the relativistic Lorentz factor associated with the phase velocity of the plasma wave $v_p$, and $\beta _p=v_p/c$. The plasma wavenumber is defined as $k_p=\omega _p/v_p$ with the plasma frequency given by $\omega _p=\sqrt{n_0 e^2/(m_e\varepsilon _0)}$. The longitudinal electric field, $E_z$, and electron density modulation, $\delta n/n_0$ are given as:

2 $$\begin{aligned} \frac{E_{z}}{E_{0}}&= -\frac{1}{k_{p}} \frac{\partial \phi }{\partial \zeta } \end{aligned}$$

3 $$\begin{aligned} \frac{\delta n}{n_{0}}&= \frac{1}{k_{p}^{2}} \frac{\partial ^{2} \phi }{\partial \zeta ^{2}}, \end{aligned}$$

where $E_0=m_e c \omega _p/e$ is the wave breaking field that corresponds to the maximum possible electric field of the plasma wave. The above equations are solved to estimate the plasma response to the pre-pulse, and the corresponding numerical results are illustrated in the Supplementary Fig. S2, where the position of the peak of the driver pulse is chosen to be $0.85\ \lambda _p$ from the position of pre-pulse. We have also performed a set of simulations for different delay lengths between the pulses. From the analysis of the simulations, we conclude stable propagation of the driver laser pulse for delay lengths between 0.85 $\lambda _p$ and 0.875 $\lambda _p$ (see section S2 of the Supplementary Information for details). As an initial value to begin optimization we suggest choosing the peak of the driver pulse positioned ahead of the density peak driven by the pre-pulse.

## Supplementary Information

Supplementary Information 1.

## Data Availability

The datasets generated and/or analysed during the current study are available in the University of Strathclyde KnowledgeBase repository, https://doi.org/10.15129/8301b69c-8a57-40e1-a29d-3f8d5832f316.
