# Supplementary material for: Propagation of intense laser pulses in plasma with a prepared phase-space distribution
Source: Sci Rep. 2022 Nov 27;12:20368. doi: 10.1038/s41598-022-24664-x (PMC9701684; doi:10.1038/s41598-022-24664-x)
Supplement: Supplementary file 1 — Supplementary Information 1. [file 41598_2022_24664_MOESM1_ESM.pdf]

Supplementary Information:
Propagation of intense laser pulses in plasma with a prepared phase-space distribution

Devki Nandan Gupta,[1] Samuel R. Yoffe[†],[2] Arohi Jain,[1] Bernhard Ersfeld,[2] and Dino A. Jaroszynski[*,2]

[1] *Department of Physics and Astrophysics, University of Delhi, Delhi 110 007, India*

[2] *Department of Physics, SUPA & University of Strathclyde, Glasgow G4 0NG, United Kingdom*

[†]Corresponding author: sam.yoffe@strath.ac.uk

[*]Corresponding author: d.a.jaroszynski@strath.ac.uk

S1. EFFECT OF THE PRE-PULSE

The effect of the pre-pulse on the electron density modulation $\delta n/n_0$, transverse electron momentum $p_x/(m_e c)$, and longitudinal electron momentum $p_z/(m_e c)$ in plasma without the driver pulse is shown in Supplementary Fig. S1. The dashed line (- - -) indicates the position chosen for the peak of the driver laser pulse, ahead of the back of the wake and in the converging streams of electrons displaced by the pre-pulse. These electrons have both forwards (longitudinal) and *inward* radial momenta, as shown in Supplementary Fig. S1(a) and (b), respectively. The driver pulse encounters a reduced on-axis electron density [part (c)] and interacts with the converging electrons, driven by pre-pulse, to produce a well-defined parabolic plasma channel that facilitates stable propagation of the second pulse, as shown in Fig. 1(b) of the main manuscript.

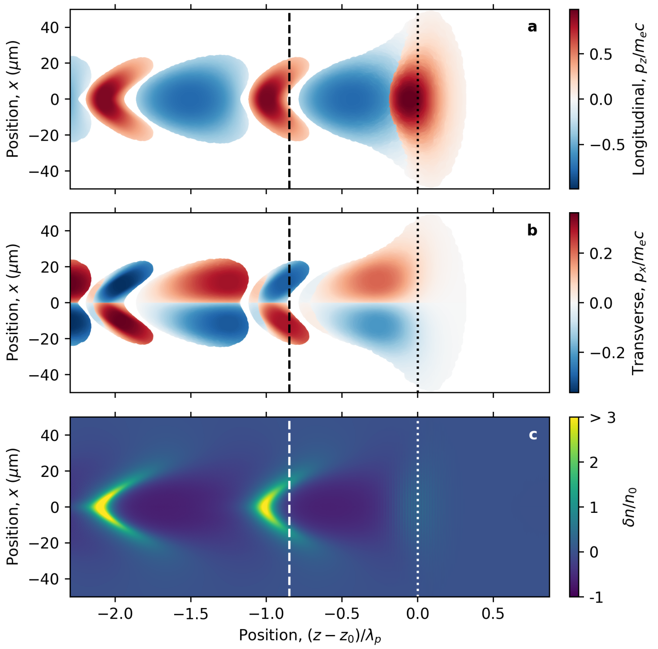

FIG. S1. The weakly nonlinear wake produced by the pre-pulse. High density electrons converging at the rear of the wake have (a) positive longitudinal momentum, $p_z > 0$, and (b) inward transverse momentum, $p_x/x < 0$, when they are deflected by the driver pulse and split to form the narrow plasma channel observed in Fig. 1 of the main manuscript. The position of the peak of the pre-pulse, z_0 ($\cdots$) and driver pulse (- - -) are indicated. Part (c) shows the electron density modulation $\delta n/n_0$, where increased density is observed for the converging electron streams. This snapshot using only the pre-pulse laser is taken at $ct = 4\,\mathrm{mm}$, where the normalized amplitude of the pre-pulse is 1.85.

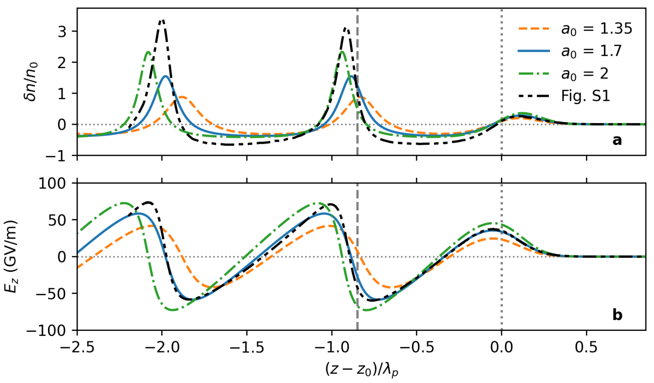

FIG. S2. Nonlinear theory[1] used to estimate the plasma response to the pre-pulse laser. Part (a) shows the density modulation, $\delta n/n_0$, and part (b) the longitudinal electric field, E_z, with z_0 the position of the intensity peak of the pre-pulse laser and λ_p the plasma wavelength. The vertical dashed line indicates the delay chosen in this work, where good channel production and stable propagation is observed when $a_0 \gtrsim 1.7$. The on-axis density and E_z line-out from Fig. S1 ($\cdot\cdot -$), where $a_0 = 1.85$, are shown for comparison. This figure is for a plasma density of $n_0 = 7.17 \times 10^{17}\,\mathrm{cm}^{-3}$.

S2. ESTIMATING OPTIMUM DELAY

For optimizing delay between pre-pulse and driver pulse, we use a nonlinear 1D theory[1,2] with the plasma and pre-pulse parameters used in the main manuscript. The Eqs. (1)–(3) of the main manuscript are solved to estimate the plasma response to the pre-pulse, and the corresponding numerical results are illustrated in Supplementary Fig. S2. The dashed line (- - -) is chosen as the position of the peak of the driver pulse, which is at a distance of 0.85 λ_p from the position of pre-pulse ($\cdots$). The plasma wave driven by the pre-pulse is not directly used to guide the driver pulse, nor is the delay tuned to resonantly excite the plasma wave. For the parameters used here, we found that reducing the delay to 0.85 λ_p caused the oscillating electrons to interact with the driver pulse as they travel back towards the laser axis as a denser sheath current, resulting in a narrow high-walled density channel surrounding the laser. Observation from simulations show that this is a parabolic channel that can be used as a waveguide to sustain high-intensity ($I_{rms} > 4.5 \times 10^{19}\,\mathrm{W/cm^2}$) pulses over nearly a centimetre in low density plasma. If longer pulse delays are required then, provided that the pre-pulse intensity remains sufficiently low to generate a well-behaved plasma wave below the threshold for self-injection, suitable plasma conditions could also be found in subsequent plasma oscillation cycles (bubbles).

To give the reader a qualitative understanding of the delay chosen for stable propagation of the main driver pulse, we have performed a set of simulations for different delay lengths between the pulses. Fig. S3 shows the transverse electron density profile at the position of peak intensity of the driver pulse at delay lengths [0.5, 0.75, 0.8, 0.825,0.85,0.875, 0.9, 1]$\times\lambda_p$ in panels (a)-(h), respectively. For the same delay lengths, Fig. S4 shows the time evolution of the on-axis longitudinal electric field, E_z at $r = 0$, in the moving window, $\zeta = z - ct$, where the dotted lines indicate the center point of the bubble. When the position of the back of the bubble moves forward in the moving window it is superluminal. The back and center of the bubble fluctuate significantly for delays $> 0.9\ \lambda_p$ and $< 0.825\ \lambda_p$. The plots in Fig. S3 show that when the charge flows and converges on the head of the main laser pulse it is deflected and produces a narrow parabolic channel, as shown in panels (e)-(f). The corresponding field evolution at the center and back of the bubble is also stable as shown in Fig. S4 (e)-(f). For a short delay ($< 0.8\ \lambda_p$), electrons do not converge at the head of the driver pulse (which only experiences very low electron density) and cannot form a channel capable of guiding the pulse as shown in Figs. S3 (a)-(b). In contrast, for longer delays ($> 0.9\ \lambda_p$) the converging charge crosses the laser axis ahead of the driver pulse and therefore does not provide the required inward momenta necessary to balance the ponderomotive force to produce the channel, as shown in Figs. S3(h). These simulations confirm that an optimum delay is achieved when the main driver laser pulse is ahead of the "back of the pre-pulse bubble", which ensures that electron streams converge on the pulse with sufficient inward momenta to create the channel.

The production of a co-moving parabolic density channel is not sufficient to claim the stable propagation of the main laser pulse. Figure S5 shows the temporal evolution of the laser amplitude and waist for a range of delay lengths. The top two panels show the main laser pulse, which is observed to self-focus in the plasma before diffracting. A stable and constant laser amplitude is observed for delays between 0.85 λ_p and 0.875 λ_p. The lower two panels show the evolution of the pre-pulse. When the delay is very short (0.5 λ_p) the pulses interfere (through their interaction with the plasma). This parameter scan is in full agreement with our understanding and description of the channel

production and pulse guiding mechanisms.

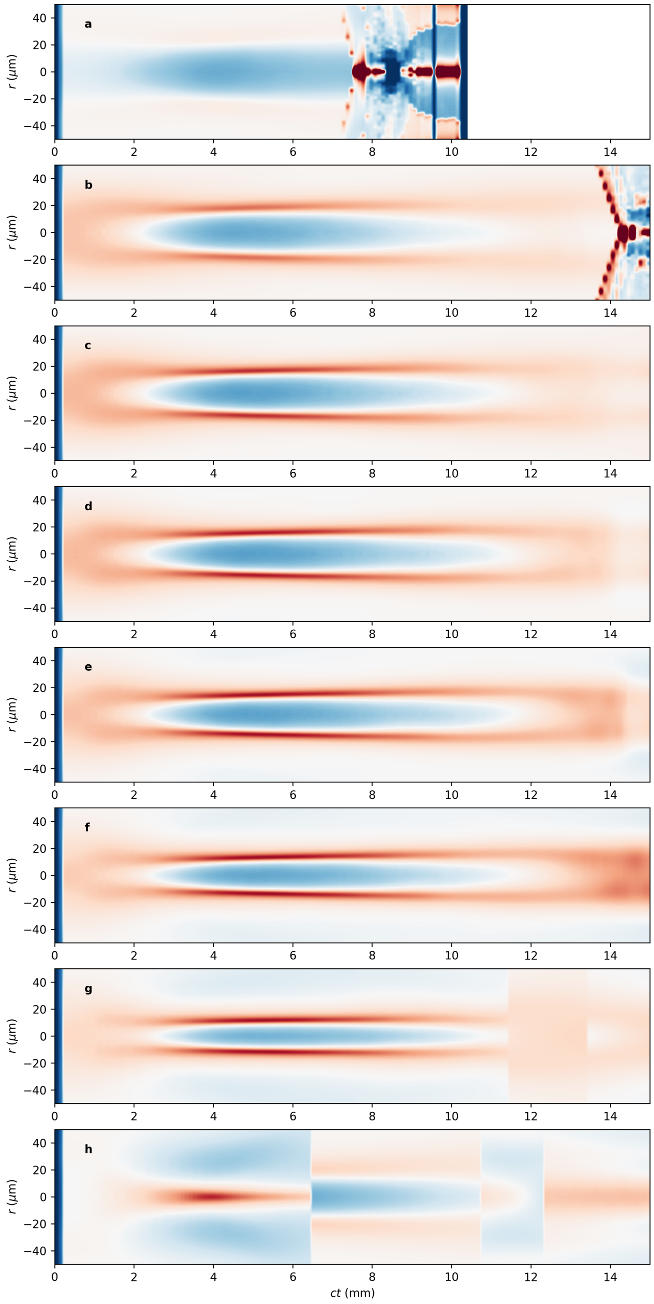

FIG. S3. Evolution of the transverse electron density profile, determined at the position of peak intensity of the driver pulse, for the delay lengths [0.5, 0.75, 0.8, 0.825,0.85,0.875, 0.9, 1] λ_p in panels (a)-(h), respectively. Panel (e)-(f) demonstrates the production of a persistent plasma channel, with steep walls and a low density core.

[1] Esarey, E., Schroeder, C. B. & Leemans, W. P. Physics of laser-driven plasma-based electron accelerators. *Reviews of Modern Physics* **81**, 1229 (2009).

[2] Sprangle, P., Esarey, E. & Ting, A. Nonlinear theory of intense laser-plasma interactions. *Phys. Rev. Lett.* **64**, 2011–2014 (1990).

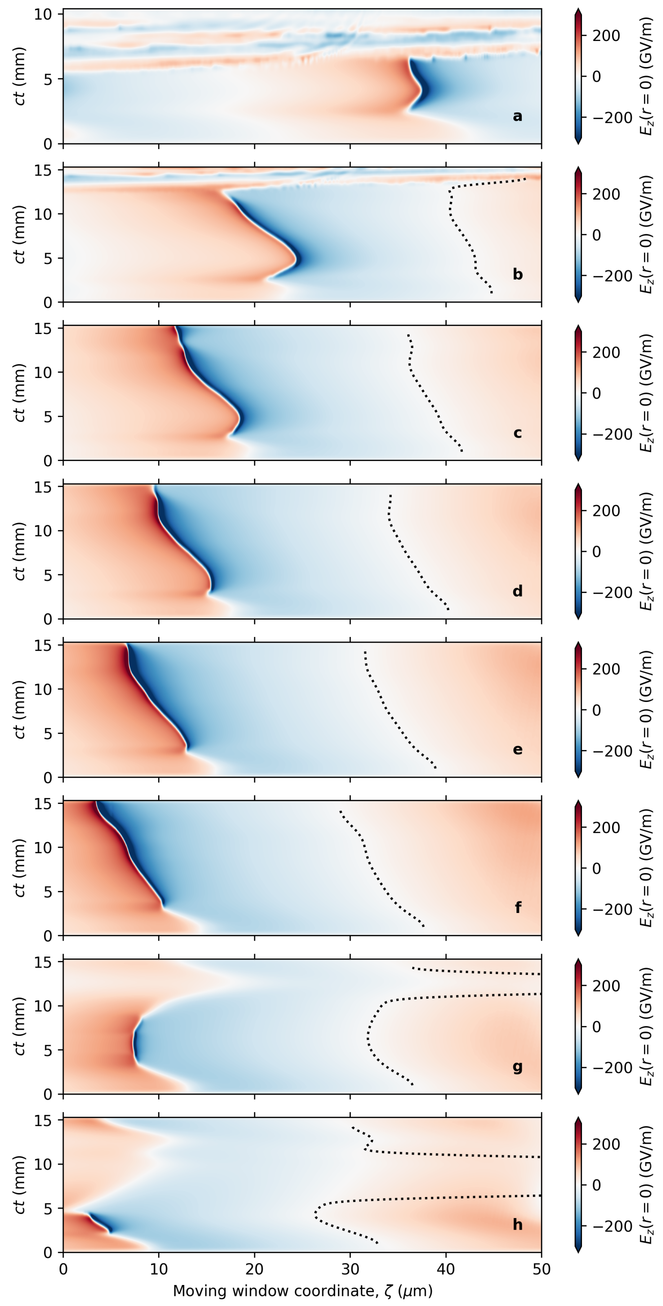

FIG. S4. Temporal evolution of the on-axis longitudinal electric field at delay lengths [0.5, 0.75, 0.8, 0.825,0.85,0.875, 0.9, 1] λ_p in panels (a)-(h),respectively, shown in the moving window, $\zeta = z - ct$. Dotted lines indicate the position of the dephasing point.

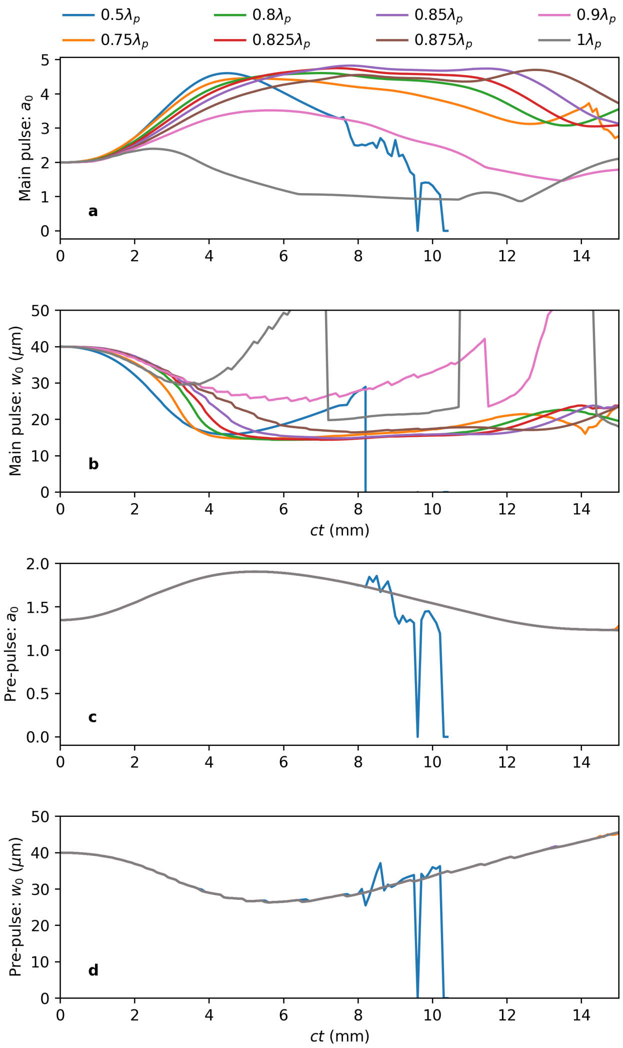

FIG. S5. Evolution of the driver pulse (a) amplitude and (b) waist, for different delay lengths. A region of stable driver pulse guiding is identified for delay lengths 0.85 λ_p and 0.875 λ_p. Evolution of pre-pulse amplitude and waist is shown in panels (c) and (d), respectively.
